# Gut feelings: the relations between depression, anxiety, psychotropic drugs and the gut microbiome

**DOI:** 10.1080/19490976.2023.2281360

**Published:** 2023-11-28

**Authors:** S. Brushett, R. Gacesa, A. Vich Vila, M.F. Brandao Gois, S. Andreu-Sánchez, J.C. Swarte, M.A.Y. Klaassen, V. Collij, T. Sinha, L.A. Bolte, J. Wu, M. Swertz, M.L.A. de Kroon, S.A. Reijneveld, C. Wijmenga, R.K. Weersma, J. Fu, H.M. van Loo, A. Kurilshikov, A. Zhernakova

**Affiliations:** a Department of Genetics, University of Groningen and University Medical Center Groningen, Groningen, The Netherlands; b Department of Health Sciences, University of Groningen and University Medical Center Groningen, Groningen, The Netherlands; c Department of Gastroenterology and Hepatology, University of Groningen and University Medical Center Groningen, Groningen, The Netherlands; d Department of Microbiology and Immunology, Rega Institute for Medical Research, Leuven, Belgium; e VIB-KU Leuven Center for Microbiology, Leuven, Belgium; f Department of Pediatrics, University of Groningen and University Medical Center Groningen, Groningen, The Netherlands; g Genomics Coordination Center, University of Groningen, University Medical Center Groningen, Groningen, The Netherlands; h Department of Psychiatry, Interdisciplinary Center Psychopathology and Emotion regulation, University of Groningen, University Medical Center Groningen, Groningen, The Netherlands

**Keywords:** gut-brain axis, gut microbiome, internalizing disorders, major depressive disorder, dysthymia, generalized anxiety disorder, psychotropic drugs

## Abstract

The gut microbiome is involved in the bi-directional relationship of the gut – brain axis. As most studies of this relationship are small and do not account for use of psychotropic drugs (PTDs), we explored the relations of the gut microbiome with several internalizing disorders, while adjusting for PTDs and other relevant medications, in 7,656 Lifelines participants from the Northern Netherlands (5,522 controls and 491 participants with at least one internalizing disorder). Disorders included dysthymia, major depressive disorder (MDD), any depressive disorder (AnyDep: dysthymia or MDD), generalized anxiety disorder (GAD) and any anxiety disorder (AnyAnx: GAD, social phobia and panic disorder). Compared to controls, 17 species were associated with depressive disorders and 3 were associated with anxiety disorders. Around 90% of these associations remained significant (FDR <0.05) after adjustment for PTD use, suggesting that the disorders, not PTD use, drove these associations. Negative associations were observed for the butyrate-producing bacteria *Ruminococcus bromii* in participants with AnyDep and for *Bifidobacterium bifidum* in AnyAnx participants, along with many others. Tryptophan and glutamate synthesis modules and the 3,4-Dihydroxyphenylacetic acid synthesis module (related to dopamine metabolism) were negatively associated with MDD and/or dysthymia. After additional adjustment for functional gastrointestinal disorders and irritable bowel syndrome, these relations remained either statistically (FDR <0.05) or nominally (*P* < 0.05) significant. Overall, multiple bacterial species and functional modules were associated with internalizing disorders, including gut – brain relevant components, while associations to PTD use were moderate. These findings suggest that internalizing disorders rather than PTDs are associated with gut microbiome differences relative to controls.

## Introduction

The role of the gut – brain axis and the involvement of the gut microbiome in the onset and pathogenesis of internalizing disorders (e.g. depression and anxiety) is an emerging field of study.^1^ The gut and brain exhibit a bi-directional relationship with multiple routes of communication, including the immune system, the enteric nervous system, the vagus nerve and the hypothalamic – pituitary–adrenal axis.^1^ The gut microbiome is the ecosystem of microbes that inhabit the human gut, and it has been associated with various health- and disease-related traits, including mental disorders.^2–4^ Gut microbes exhibit a range of metabolic activities that have potential effects on the brain. For example, short-chain fatty acids (SCFAs) produced by gut bacteria promote indirect signaling to the brain by stimulating enteroendocrine cells to produce gut hormones.^5^ Gut bacteria can also produce neurotransmitters and their precursors, including serotonin, tryptophan, norepinephrine and γ-aminobutyric acid.^5,6^ Several *in vivo* and intervention studies, such as probiotic supplementation and fecal microbiota transplantation studies in animals and humans, have suggested a causal relationship between the gut microbiome and internalizing disorders.^1,7–14^

While there is evidence for a microbiota – gut–brain axis, current studies of internalizing disorders and the gut microbiome in humans are limited in power due to their relatively small sample sizes, leading to inconsistent findings.^8^ Another common limitation is the use of the 16S rRNA sequencing technique to profile microbiome taxonomy, even though this technique has limited taxonomic and functional resolution compared to the metagenomic shotgun sequencing approach. In addition, most studies do not account for the confounding effects of medication or functional gastrointestinal disorders (FGIDs) and irritable bowel syndrome (IBS) in their analyses,^8^ even though these factors are known to influence the gut microbiome and IBS is associated with internalizing disorders.^2,4,8,15–18^ Previous studies also mainly included participants with major depressive disorder (MDD) and generalized anxiety disorder (GAD),^8^ while the relations of the gut microbiome with dysthymia have not yet been studied.

**Figure 1.**
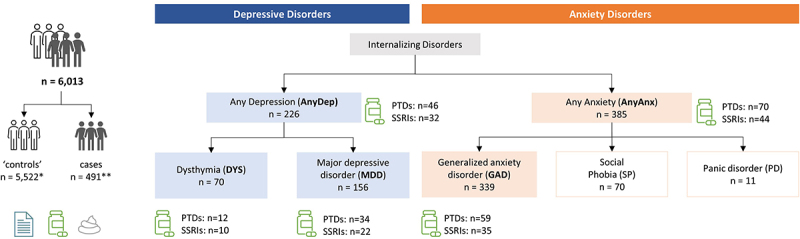
Schematic of study design.

In this study we aimed to explore the associations of the gut microbiome with several internalizing disorders, with adjustment for use of psychotropic drugs (PTDs). The internalizing disorders we examined included depressive disorders (dysthymia, MDD and any depressive disorder (AnyDep: dysthymia or MDD)) and anxiety disorders (GAD and any anxiety disorders (AnyAnx: GAD, social phobia and panic disorder)). The number of cases per disorder ranged from 70 to 385 (Figure 1). In total, we analyzed data from 7,656 participants of the Lifelines population cohort, which comprises Dutch individuals from the northern provinces of the Netherlands.^19^

This cross-sectional study explored the relationship between several internalizing disorders (range: 70–385 cases), psychotropic drug (PTD) use and the gut microbiome. Data on internalizing disorders and medication use was available from around the time of stool sample collection. The disorders in white boxes were not analyzed individually. *Controls included participants (*n* = 5,522) without any internalizing disorders. **One or more internalizing disorders were observed in 491 participants (see Figure S1 for more details).

## Results

### Cohort description

To study the relations between depression, anxiety, use of PTDs and the gut microbiome, we first assessed participants for any internalizing disorders and PTD use (Figure 1). Of the 7,656 participants investigated, 156 had MDD, 70 had dysthymia and 339 had GAD (Figure 1, Table S1). Overall, 226 participants had AnyDep (inclusive of MDD and dysthymia) and 385 had AnyAnx (inclusive of GAD or social phobia or panic disorder). In this study, 491 participants had at least one internalizing disorder (Supplementary Results; Figure S1). PTDs (selective serotonin-reuptake inhibitors (SSRIs), tricyclic antidepressants and other psychoanaleptics, benzodiazepine agonists and other psycholeptics and antipsychotics) were used by 373 (5%) participants, including 165 (44.2%) control participants (participants without any internalizing disorders (Table S1)). PTD use by controls could reflect: 1) previously diagnosed participants who no longer presented with (as many) symptoms but were still receiving medication, 2) participants who were receiving medication for a condition other than a mental illness, for example IBS,^20^ or 3) participants who were receiving medication for an internalizing disorder diagnosed via an alternative route to the DSM-IV-TR MINI^21^ criteria that was used in this study.

We next compared several factors in participants with (*n* = 491) and without internalizing disorders (defined as controls; *n* = 5,522) to adjust for potential confounders in subsequent analyses (Table S1). Mean diet quality score, inflammatory bowel disease (IBD, including Crohn’s disease (CD) and ulcerative colitis (UC)) and antibiotic use of participants with internalizing disorders were not significantly different in all cases in comparison to controls (*P* > 0.05). Age, sex, body mass index (BMI), proton pump inhibitor use (PPIs), PTD use, any FGIDs and IBS were significantly different for most disorders (*P* > 0.05). Thus, the main analysis was first adjusted for age, sex, BMI, PPIs and PTDs (either SSRI or any PTD use) and thereafter additionally confirmed to not be influenced by diet and antibiotics by comparing models with and without these two parameters (see ‘*Taxonomic, pathway and gut – brain module associations’* in Methods for more details). We then further investigated bacterial taxa (Table S2), pathways (Table S3) and gut – brain module (GBM; Table S4) associations by adjusting for any FGIDs and IBS.

### Internalizing disorders are moderately associated with overall gut microbiome composition

We first explored associations between internalizing disorders, with and without adjustment for PTD use (SSRIs or any PTDs), and the gut microbiome diversity within participants (alpha diversity) and between participants (beta diversity). Overall, no significant associations were observed between internalizing disorders or PTDs and alpha diversity (false discovery rate (FDR)>0.05, Tables S5–7). All internalizing disorders moderately (R^2^_adj_ values) but significantly explained the gut microbiome variation between participants, whether adjusted or unadjusted for SSRI or PTD use (*P*_adj_ <0.05; FDR < 0.05, Figure S2, see Table S8 for details on R^2^_adj_ values).

### Bacterial species are mostly associated with depressive disorders, independent of PTD use

**Figure 2.**
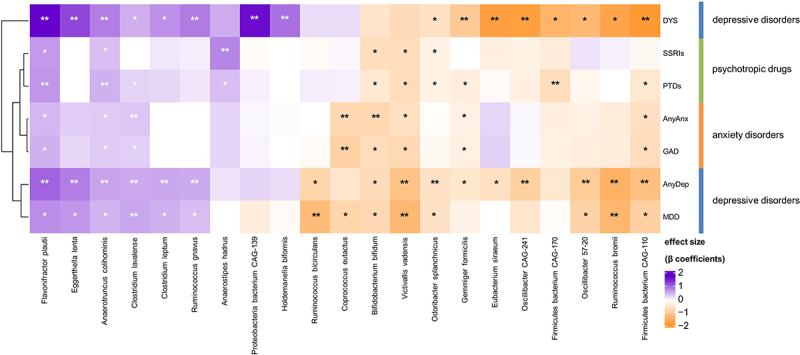
Bacterial species associated with internalizing disorders and psychotropic drugs.

**Figure 3.**
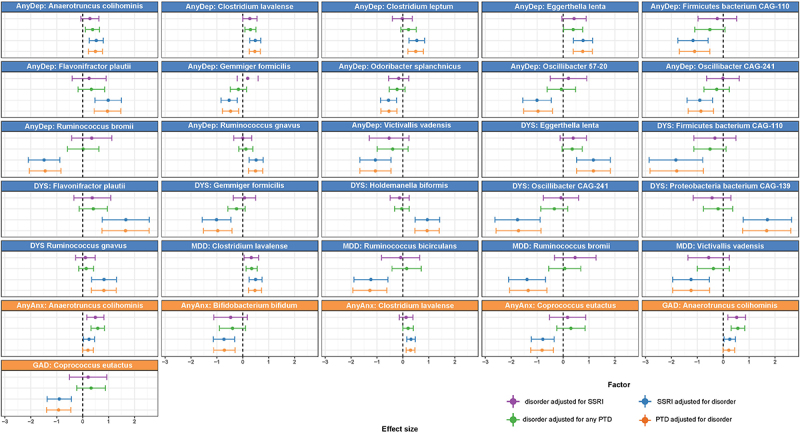
Bacterial species associated with internalizing disorders, adjusted for the use of SSRIs or of any PTDs and vice versa.

We next investigated which bacterial taxa were associated with internalizing disorders and PTDs. In the single-trait analysis (either single disorder or single drug (i.e. SSRIs or any PTDs)), we identified 79 significant (FDR <0.05) associations to all traits at all taxonomic levels (Table S9). We also observed an overlap between some of the bacterial species that were associated with all internalizing disorders and PTDs (Figure 2). However, more species were significantly associated with internalizing disorders after adjustment for PTDs, in comparison to associations with PTDs adjusted for internalizing disorders (FDR_adj_ <0.05; Figure 3, Tables S10 and S11). For example, eight species were significantly associated with dysthymia, whether adjusted for SSRIs (Figure 3, Table S10) or any PTDs (Figure 3, Table S11), whereas only one species was associated with any PTDs (Figure 3, Table S11) and none were associated with SSRIs after adjustment (Figure 3, Table S10). Overall, we also observed that, compared to controls, more species were associated with depressive disorders (AnyDep = 13 taxa, dysthymia = 8 taxa, MDD = 4 taxa) than with anxiety disorders (AnyAnx = 3 taxa, GAD = 1 taxon; Figure 3, Tables S10 and S11). In comparison to controls, dysthymia was also associated with more changes in bacterial species than MDD (8 versus 4 taxa; Figure 3, Tables S10 and S11). At species level, after adjustment for any PTDs in participants with depressive disorders, we observed larger and significant negative effect sizes for the relative abundances of *Ruminococcus bromii* (Figure 3, Table S11; AnyDep: β=-1.4, SE = 0.3, FDR_adj_ = 0.005; MDD: β=-1.3, SE = 0.4, FDR_adj_ = 0.03), an *uncharacterized Firmicutes bacterium CAG-110* (AnyDep: β=-1.1, SE = 0.3, FDR_adj_ = 0.03; dysthymia: β=-1.8, SE = 0.5, FDR_adj_ = 0.04), *Oscillibacter species CAG-241* (AnyDep: β=-0.9, SE = 0.3, FDR_adj_ = 0.04; dysthymia: β=-1.7, SE = 0.4, FDR_adj_ = 0.02), *Victivallis vadensis* (AnyDep: β=-1.1, SE = 0.3, FDR_adj_ = 0.04; MDD: β=-1.2, SE = 0.4, FDR_adj_ = 0.04) and *Ruminococcus bicirculans* (MDD: β=-1.3, SE = 0.3, FDR_adj_ = 0.03). In contrast, we observed large, significant positive effect sizes at species level for the relative abundances of *Eggerthella lenta* (Figure 3, Table S11; AnyDep: β = 0.8, SE = 0.2, FDR_adj_ = 0.02; dysthymia: β = 1.2, SE = 0.3, FDR_adj_ = 0.04), an *uncharacterized Proteobacteria bacterium CAG-139* (dysthymia: β = 1.7, SE = 0.5, FDR_adj_ = 0.04) and *Flavonifractor plautii* (AnyDep: β = 1, SE = 0.3, FDR_adj_ = 0.03; dysthymia: β = 1.7, SE = 0.5, FDR_adj_ = 0.04), also after adjustment for any PTDs in participants with depressive disorders. After further adjusting the models adjusted for any PTDs for antibiotic use and diet quality score, all the associations remained significant (Table S12).

Heatmap depicting the effect sizes (β coefficients) of bacterial taxa associations at species level with internalizing disorders and use of any psychotropic drugs (incl. SSRI) in the unadjusted model. * indicates nominal significance (*P* < 0.05). ** indicates both nominal and FDR significance (*P* < 0.05, FDR < 0.05). Abbreviations – DYS: dysthymia, MDD: major depressive disorder, AnyDep: any depression, GAD: generalized anxiety disorder, AnyAnx: any anxiety, PTDs: all psychotropic drugs, SSRI: selective serotonin-reuptake inhibitors.

Forest plots depicting the confidence intervals of effect sizes (β ± 1.96*SE) of associations between CLR-transformed bacterial species and i) internalizing disorders adjusted for SSRIs (purple) or any PTDs (green) and ii) SSRIs (blue) and any PTDs (orange) adjusted for internalizing disorders. Abbreviations – AnyDep: any depression, DYS: dysthymia, MDD: major depressive disorder, AnyAnx: any anxiety, GAD: generalized anxiety disorder, PTDs: all psychotropic drugs, SSRI: selective serotonin-reuptake inhibitors.

Although the overall pattern and direction of association of bacterial species with internalizing disorders and PTD use appeared to be similar (Figure 2, Table S9), after adjustment for PTDs we observed individual associations of many bacterial species with specific internalizing disorders (Figure 3, Tables S10 and S11). For example, we observed negative associations of AnyDep with *R. bromii* and two *Oscillibacter* species and of MDD with *R. bicirculans* and *R. bromii* that were independent of the effects of PTDs, as were several other associations (Figure 3, Tables S10 and 11). After adjustment for PTDs, depressive disorders (especially dysthymia) were associated with more differences in bacterial taxa compared to controls than were anxiety disorders, even though there was a larger number of participants with anxiety disorders. Overall, PTDs were only moderately associated with bacterial species in comparison with internalizing disorders.

### Bacterial pathways and gut–brain modules are associated with depressive disorders, independent of PTD use

Following taxa associations, we next investigated the functional potential of the gut microbiome by exploring associations with bacterial metabolic pathways and gut – brain modules (GBMs). Metabolic pathways were annotated using the MetaCyc database and included 164 genetic microbial pathways present in > 5% of participants.^22,23^ GBMs comprise curated modules of microbial pathways involved in the metabolism of molecules with the potential to interact with the human nervous system,^3^ and we included the 36 GBMs present in > 5% of participants in the analysis. For bacterial metabolic pathways, the most significant associations were observed for AnyDep and dysthymia (FDR <0.05, Tables S13–15), with the associated pathways mainly involved in sugar metabolism and synthesis of vitamins and amino acids. Interestingly, AnyDep, MDD and GAD were nominally (*P* < 0.05) associated with a moderate decrease in the relative abundance of the glutamine and glutamate biosynthesis pathway, after adjustment for SSRIs or any PTDs (Tables S14 and S15; SRRIs and any PTDs: AnyDep: β=-0.1, SE = 0.04, *P*_adj_ = 0.01; MDD: β=-0.1, SE = 0.05, *P*_adj_ = 0.01; GAD: β=-0.1, SE = 0.03, *P*_adj_ = 0.04). Both components are involved in the microbiota – gut–brain axis and have been implicated in mood disorders, including MDD.^1,24,25^

**Figure 4.**
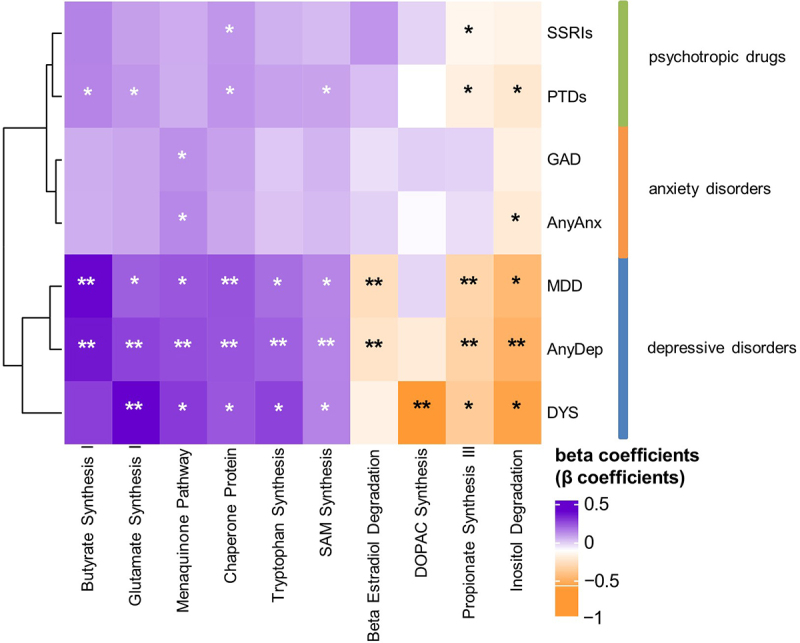
Bacterial gut – brain modules associated with internalizing disorders and psychotropic drugs.

**Figure 5.**
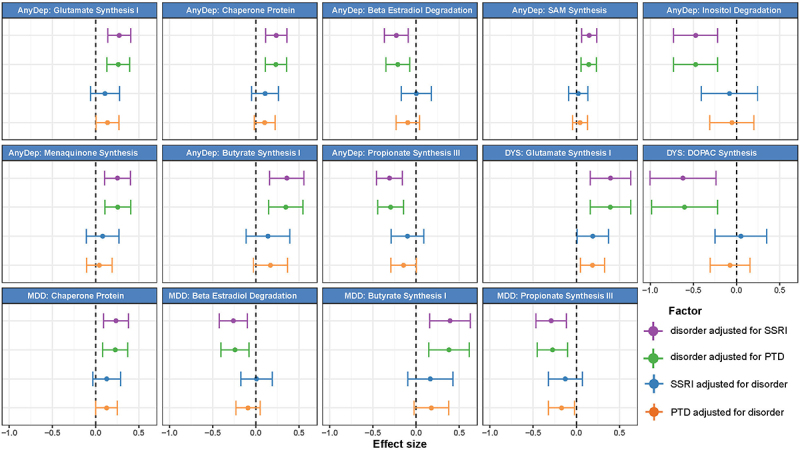
Bacterial gut – brain modules associated with internalizing disorders, adjusted for the use of SSRIs or of any PTDs and vice versa.

We then examined associations between internalizing disorders and GBMs. Of the 36 GBMs investigated, 10 were significantly associated with depressive disorders before adjusting for PTDs (Figure 4, Table S16), 9 remained significant after adjusting for any SSRIs (Figure 5, Table S17) and 7 remained significant after adjusting for any PTDs (Table S18, Figures 5). Overall, we did not find any significant (FDR <0.05) association between GBMs and anxiety disorders or PTD use. In the unadjusted models, AnyDep and dysthymia were significantly associated with an increase in glutamate synthesis I (Figure 4, Table S16; AnyDep: β = 0.3, SE = 0.1, FDR_adj_ = 0.01; dysthymia: β = 0.4, SE = 0.1, FDR_adj_ = 0.04). Dysthymia was significantly associated with a decrease in 3,4-Dihydroxyphenylacetic acid (DOPAC) synthesis after adjustment for SSRI use (Figure 5, Table S17; β=-0.6, SE = 0.2, *P*_adj_ = 0.002, FDR_adj_ = 0.03), while a nominally significant association remained after adjustment for any PTD use (Table S18; β=-0.6, SE = 0.2, *P*_adj_ = 0.002, FDR_adj_ = 0.06). Notably, DOPAC synthesis was previously associated with good quality of life.^3^ Depressive disorders were also distinctly associated with SCFAs. Positive associations were observed with butyrate synthesis I when adjusted for SSRIs (Figure 5, Table S17; AnyDep: β = 0.4, SE = 0.1, FDR_adj_ = 0.02; dysthymia: β = 0.4, SE = 0.1, FDR_adj_ = 0.04) and any PTDs (Figure 5, Table S18; AnyDep: β = 0.3, SE = 0.1, FDR_adj_ = 0.02; dysthymia: β = 0.4, SE = 0.1, FDR_adj_ = 0.04). In contrast, negative associations were observed for propionate synthesis III when adjusted for SSRIs (Figure 5, Table S17; AnyDep: β=-0.3, SE = 0.1, FDR_adj_ = 0.01; MDD: β=-0.3, SE = 0.1, FDR_adj_ = 0.03) and any PTDs (Figure 5, Table S18; AnyDep: β=-0.3, SE = 0.1, FDR_adj_ = 0.01). All depressive disorders were also nominally associated with a moderate increase in tryptophan synthesis when adjusted for SSRIs (Table S17; AnyDep: β = 0.2, SE = 0.1, *P*_adj_ = 0.003, FDR_adj_ = 0,05; dysthymia: β = 0.3, SE = 0.1, *P*_adj_ = 0.03, FDR_adj_ = 0.3; MDD: β = 0.2, SE = 0.1, *P*_adj_ = 0.03, FDR_adj_ = 0.3) and any PTDs (Table S18; AnyDep: β = 0.2, SE = 0.1, *P*_adj_ = 0.005, FDR_adj_ = 0,08; dysthymia: β = 0.3, SE = 0.1, *P*_adj_ = 0.03, FDR_adj_ = 0.3; MDD: β = 0.2, SE = 0.1, *P*_adj_ = 0.04, FDR_adj_ = 0.3). After further adjusting the models adjusted for any PTDs for antibiotic use and diet quality score, all the previously significant associations with bacterial pathways and GBMs remained significant (Table S19 and S20). Overall, these results provide mechanistic clues about the relationship of the gut microbiome with internalizing disorders, especially depressive disorders, via components known to be involved in the microbiota – gut–brain axis.

Heatmap depicting the effect sizes (β coefficients) of bacterial gut – brain modules associations with internalizing disorders and use of any psychotropic drugs (incl. SSRI) in the unadjusted model. * indicates nominal significance (*P* < 0.05). ** indicates both nominal and FDR significance (*P* < 0.05, FDR < 0.05). Abbreviations – DYS: dysthymia, AnyDep: any depression, MDD: major depressive disorder, AnyAnx: any anxiety, GAD: generalized anxiety disorder, PTDs: all psychotropic drugs, SSRI: selective serotonin-reuptake inhibitors, SAM: S-Adenosyl Methionine, DOPAC: 3,4-Dihydroxyphenylacetic acid.

Forest plots depicting the confidence intervals of effect sizes (β ± 1.96*SE) of associations between CLR-transformed bacterial gut – brain modules and i) internalizing disorders adjusted for SSRIs (purple) or any PTDs (green) and ii) SSRIs (blue) and any PTDs (orange) adjusted for internalizing disorders. Abbreviations – AnyDep: any depression, DYS: dysthymia, MDD: major depressive disorder, PTDs: all psychotropic drugs, SSRI: selective serotonin-reuptake inhibitors, SAM: S-Adenosyl Methionine, DOPAC: 3,4-Dihydroxyphenylacetic acid.

### After adjustment for FGIDs and IBS, most relations between internalizing disorders and the gut microbiome remain FDR (<0.05) or nominally (P < 0.05) significant

Lastly, given that internalizing disorders tend to be associated with FGIDs and IBS,^18^ we further adjusted all analyses for these factors (Tables S21-S32). No new associations were observed, but several associations remained after these additional adjustments. For taxa associations, 30% (*n* = 9) of species associations adjusted for SSRIs remained (Table S22), while 29.6% (*n* = 8) of associations adjusted for any PTDs remained (Table S23). Overall, the most significant associations (highest FDRs) from the main analyses remained significant (Tables S10 and S11), and the associations that were no longer FDR-significant were still nominally significant (*P* < 0.05) (Tables S21-S24). Notably, associations between bacterial species and AnyAnx were lost, whether adjusted for SSRIs or any PTDs (Tables S22 and S23). For pathway associations, 15.4% (*n* = 2) of associations adjusted for SSRIs remained (Table S26), while 42.9% (*n* = 3) of associations adjusted for any PTDs remained (Table S27). The pathways that did remain significant were involved in purine nucleotide degradation and the TCA cycle (Tables S26 and S27). For the GBM associations, 35.7% (*n* = 5) of associations adjusted for SSRIs (Table S29) and 33.3% (*n* = 3) adjusted for any PTDs remained (Table S30). GBMs involved in SCFA synthesis and glutamate synthesis remained significant, whether adjusted for SSRIs (Table S29) or any PTDs (Table S30). Notably, negative associations with DOPAC synthesis were no longer FDR-significant in the follow-on analyses, but remained nominally significant when associated with dysthymia, whether adjusted for SSRIs (Table S29; β=-0.6, SE = 0.2, *P*_adj_ = 0.002, FDR_adj_ = 0.06) or any PTDs (Table S30; β=-0.6, SE = 0.2, *P*_adj_ = 0.002, FDR_adj_ = 0.07).

Overall, adjustment of the main analyses to account for FGIDs and IBS resulted in no new associations, with previous associations remaining either FDR-significant (<0.05) or nominally significant (*P* < 0.05). This suggests that the gut microbiome associations with depressive and anxiety disorders we observed were neither spurious nor solely driven by co-occurrence with FGID and/or IBS.

## Discussion

In this study, we investigated the relationship between the gut microbiome and several internalizing disorders and PTDs in a large number of participants (*n* = 7,656). We aimed to explore if internalizing disorders were associated with differences to gut microbiome composition and functionality, independent of PTD use.

One of the main challenges in investigating the relationship between internalizing disorders and the gut microbiome is accounting for the effects of PTDs.^8^ In our study, not all participants with internalizing disorders were taking PTDs, and vice versa, making it possible for us to disentangle these effects. We show that internalizing disorders, especially depressive disorders, were associated with differences in gut microbiome composition, bacterial functional pathways and gut – brain modules involved in the gut – brain axis and that these associations remained after adjustment for PTDs. We also show that, after additional adjustments for FGIDs and IBS, these associations remained either statistically (FDR <0.05) or nominally (*P* < 0.05) significant. We also observe several relevant associations of internalizing disorders with bacterial species. *Ruminococcus bromii*, a well-known SCFA-producing bacterium, was negatively associated with AnyDep and MDD. Animal and human studies have demonstrated decreased SCFA levels in depression,^26–28^ and mouse studies have shown alleviating effects on depression and anxiety symptoms in response to butyrate and SCFA supplementation.^29,30^ AnyDep and dysthymia (which has not been studied in this context) were positively associated with bacterial species previously associated with gastrointestinal diseases and other disorders, including *Eggerthella lenta* and *Flavonifractor plautii*. ^3,4,31^ Notably, only the association of *E. lenta* with AnyDep remained FDR-significant after additional adjustment for FGIDs and IBS. Nevertheless, the *Eggerthella* genus was also previously associated with depressive symptoms in a large 16S rRNA-based study (*n* = 1,054, validation cohort: *n* = 1,539).^32^ In our study, AnyDep and dysthymia were negatively associated with *Oscillibacter* species, a common gut commensal recently shown to be associated with health.^33^ However, these associations remained only nominally significant (*P* < 0.05) after the additional adjustment for FGIDs and IBS. Bacterial pathways involved in glutamate synthesis, a well-described excitatory neurotransmitter that can transfer intestinal sensory signals to the brain via the vagus nerve, were decreased in dysthymia, MDD and GAD.^34^ We also observed a reduction in the DOPAC synthesis module in participants with dysthymia. DOPAC synthesis pathways were previously shown to be positively associated with mental quality of life.^3^ Again, however, these pathway and module associations only remained nominally significant after further adjustment for FGID and IBS. We also show that depressive disorders (AnyDep, dysthymia and MDD) are nominally associated with reduced tryptophan synthesis modules, and tryptophan is a known neurotransmitter precursor^35^ previously shown to be reduced in people with MDD.^36^

The associations of gut microbiome features with PTD use, on the other hand, were moderate. After adjusting for internalizing disorders (specifically AnyAnx or GAD), the only taxa that was significantly (positively) associated with PTD use was *Anaerotruncus colihominis*, and this association was only nominally significant after further adjustment for FGIDs and IBS. *A. colihominis* has been preliminarily identified to dampen disease severity via immune regulation in a multiple sclerosis mouse model.^37^ No bacterial functional pathways or GBMs were significantly associated with PTD use after adjustment for internalizing disorders. In conclusion, PTDs were only moderately associated with the gut microbiome and did not explain the associations of internalizing disorders to the gut microbiome.

Previous associations of internalizing disorders with the gut microbiome have been explored in several studies,^8^ but these studies did not adjust for PTD use. When comparing our results to the studies with the largest sample sizes for MDD (MDD: *n* = 156, controls: *n* = 155)^38^ and GAD (GAD: *n* = 40, controls: *n* = 36),^39^ we found some similarities. In the MDD study, the gut microbiomes of MDD participants were enriched for the relative abundance of the *Bacteroides* genus and depleted for the *Blautia* and *Eubacterium* genera.^38^ In our study, the relative abundances of the *Bacteroides* and *Blautia* genera were moderately and nominally (*P* < 0.05), but not significantly (FDR >0.05) increased, mostly in depressive disorders. No FDR or nominally significant relations were observed for the *Eubacterium* genera. The GAD study showed an increase in the relative abundance of *Escherichia-Shigella, Fusobacterium* and *Ruminococcus gnavus* in GAD participants, whereas SCFA-producing bacteria were depleted.^39^ In our study, although not significant, *Fusobacterium* and *R. gnavus* were also moderately increased in GAD participants, though the latter was significantly associated with dysthymia (when unadjusted for FGIDs and IBS). We instead identified four other species significantly associated with MDD and one associated with GAD (before further adjustment for FGIDs and IBS). The four species significantly associated with MDD were *Clostridium lavalense* (increased), *R. bicirculans* (decreased), *R. bromii* (decreased) and *V. vadensis* (decreased). The species significantly associated with GAD was *Coprococcus eutactus* (decreased). In general, the bacterial species that were decreased have previously been positively associated with health, for example *C. eutactus* was previously associated with higher quality of life.^3^ Overall, the bacteria associated with MDD or GAD in previous studies were not significantly associated with MDD or GAD in our study. These differences could be explained by study design differences (such as small sample sizes, differences in microbiome preparation and analysis, etc.) and the fact that PTDs were adjusted for in our study.

The cross-sectional design of our study limits our ability to infer causal relationships between the gut microbiome and internalizing disorders. We also acknowledge that the focus of our study was on the presence or absence of internalizing disorders and not on the severity of these disorders. However, we still observed some similarities with the findings of a recent study on the gut microbiome and depression severity.^40^ Notably, in our study, participants were not excluded according to alcohol abuse or drug use. This was because heavy alcohol consumption (defined as > 350 kcal/day for men and > 280kcal/day for women) was only applicable for 0.3% (*n* = 22) of participants in this study, and because drug use was not recorded at the time of stool collection. Another limitation of our study is that the PTDs investigated were self-reported and therefore not standardized. In addition, although we included further adjustments for FGIDs and IBS in our association analyses, our sample size was limited. As a result, we cannot conclude whether the FDR-significant associations that were lost in comparison to the main analyses (without correction for FGIDs and IBS) were due to overcorrection or indeed due to FGIDs or IBS. Future studies will need to consider the influence of specific PTDs, other than SSRIs, on the relations of internalizing disorders and the gut microbiome, and these studies should be larger in size to account for all possible confounding factors. It should also be noted that this study was performed on a general Dutch population, and differences could potentially be observed in other populations due to, e.g., differences in environment, genetics and diet. To the best of our knowledge, our study is the largest to use metagenomic sequencing to characterize relationships between the gut microbiome and internalizing disorders while also accounting for the potential confounding effects of PTDs in general. This study is further unique in that we investigated the relations of dysthymia with the gut microbiome, which had not been done before. In conclusion, we have demonstrated that both dysthymia and MDD are associated with several shifts in the gut microbiome composition, functional pathways and GBMs compared to controls, while anxiety disorders and the use of PTDs are only moderately associated with the gut microbiome.

## Methods

### Study population

This study included 7,656 participants of the Lifelines cohort study^19^ for whom metagenomic sequencing data was generated by the Lifelines Dutch Microbiome Project (DMP)^4^ and who were also assessed for internalizing disorders (see details on measurements below and in Figure S3). Lifelines is a population-based, three-generational cohort study (*n* = 167,729) that assesses the biomedical, socio-demographic, behavioral, physical and psychological factors that contribute to health and disease in the general population of the Northern Netherlands. The DMP cohort study comprised 8,208 Lifelines participants from whom stool samples were collected to investigate how genetics, exposome, lifestyle and diet shape the gut microbiome in health and disease.

### Metadata collection

During the DMP study, metadata on age, sex, BMI, self-reported medication use and self-reported IBD (including CD and UC) was recorded at the time of stool collection^4^. The validated ROME III questionnaire^41^ was used to characterize FGIDs, and participants were classified as having either no functional gastrointestinal diseases or having IBS, functional diarrhea, functional constipation or functional bloating. The latter three traits were grouped to define any FGIDs. In addition, a diet quality score was derived from dietary information acquired via a semi-quantitative food frequency questionnaire administered 4 years prior to the DMP study, as previously described.^4,19^ Self-reported data on the use of the following PTDs were available: SSRIs, tricyclic antidepressants and other psychoanaleptics, benzodiazepine agonists and other psycholeptics and antipsychotics. PTDs with smaller sample sizes in the DMP study, such as monoamine oxidase inhibitors, were grouped into ‘other psychoanaleptics’ or ‘other psycholeptics’ based on ATC codes (https://www.whocc.no/atc_ddd_index/). Any PTD use, indicative of the number of participants who reported taking any PTDs at the time of stool collection, was derived by combining all the above-mentioned medications. Since SSRIs were the most-used PTD within this group (*n* = 234 (62.7%); Table S1), we performed two separate analyses in relation to drug use: one taking only SSRIs into consideration and one including any PTDs, including SSRIs. During the stool collection period, data was also collected on the use of PPIs and antibiotics, and they were included in analyses since both medications are known to be associated with the gut microbiome.^2,15^

## Measures and procedures

### Internalizing disorders

Internalizing disorders were assessed during the Lifelines cohort study within the same timeframe as stool collection (Figure S3). MDD, dysthymia, GAD, social phobia and panic disorder were assessed according to the criteria of the Diagnostic and Statistical Manual of Mental Disorders (DSM-IV-TR) using a standardized diagnostic interview based on the Mini-International Neuropsychiatric Interview (MINI).^21^ At the time of the interview, MDD, dysthymia and GAD were assessed for their occurrence within the past 2 weeks, 2 years and 6 months, respectively, according to DSM-IV-TR criteria. Social phobia and panic disorder were assessed for their occurrence in participants during the past month. Due to the nature of the questionnaires in Lifelines, dysthymia was not assessed in participants who satisfied the criteria for MDD, resulting in missing data for dysthymia. Disability criteria were included for dysthymia and social phobia, but not for MDD, GAD and panic disorder, as these items were not assessed. Exclusion criteria for alcohol use, somatic and drug use disorders were also not considered, as heavy consumers of alcohol (>350 kcal/day for men and > 280kcal/day for women) were low (*n* = 22 (0.3%)) and drug use was not recorded in the DMP at the time of stool sample collection. For our analyses, AnyDep indicates participants who presented with MDD or dysthymia, as dysthymia was not assessed in participants with MDD. AnyAnx indicates participants who presented with GAD, social phobia and/or panic disorder.

### Stool sample collection, DNA extraction and sequencing, and profiling of microbiome composition and function

Fresh frozen fecal samples were collected from a subset of Lifelines participants during the DMP cohort study. The sample collection, DNA extraction, sequencing and data cleaning steps were described previously^4^ (for more details see Supplementary Methods). The Biobakery pipeline v3^22,23^ was used to derive the taxonomic composition of metagenomes and to profile genes encoding microbial biochemical pathways. GBMs were constructed by regrouping KEGG orthologs according to the methods described by Valles-Colomer *et al.*, 2019 and Omixer-RPM version 0.3.2^3^. Briefly, GBMs comprise curated modules of microbial pathways involved in the metabolism of molecules with the potential to interact with the human nervous system. All corresponding scripts are available at: https://github.com/GRONINGEN-MICROBIOME-CENTRE/DMP.

#### Alpha diversity analysis

To investigate microbial diversity within samples, the microbial alpha diversity of the gut microbiome, as represented by the Shannon, Simpson and Inverse Simpson diversity indices, was calculated at species taxonomic level using the *diversity* function in the R package *vegan* (v 4.0.3). Because the data was left-skewed, alpha diversity measures were inverse rank normal transformed prior to performing statistical analyses. Analysis of alpha diversity with internalizing disorders, the use of SSRIs, or the use of any PTDs was performed using multivariable linear regression with adjustment for factors known to influence the gut microbiome. These included age, sex, BMI, technical parameters (metagenomic sequencing batch number, DNA concentration (ng/µl) and read depth), stool frequency and consistency (assessed by Bristol Stool Scale) and PPI use.^4^ Alpha diversity associations with internalizing disorders were additionally adjusted for the use of SSRIs or any PTDs. Similarly, alpha diversity associations with SSRIs or any PTDs were also adjusted for internalizing disorders (for more details see Supplementary Methods).

#### Beta diversity

Beta diversity was calculated using the Bray-Curtis distance matrix with the relative abundance of microbial species. To determine the amount of variation in beta diversity that could be explained by internalizing disorders and/or PTD use (SSRIs only or any PTDs), we implemented permutational multivariate analysis of variance in the *adonis* function of the *vegan* package in R (v 4.0.3), using 1000 permutations. Here, we adjusted for the same factors that we adjusted for in the alpha diversity multivariable linear regression analysis (for more details see Supplementary Methods). Principle coordinate analysis was visualized using the *vegdist* function (with Bray-Curtis distance) of the *vegan* package in R and the *cmdscale* function in base R.

#### Taxonomic, pathway and GBM associations

To determine which microbial taxa, biochemical pathways and GBMs were associated with a particular determinant, we used the same multivariable linear models described above to investigate the associations between these bacterial features and each internalizing disorder and the use of SSRIs or any PTDs. Microbiome features significantly associated with internalizing disorders when adjusted for any PTDs were further adjusted for antibiotic use and diet quality. These main analyses were further investigated by adjusting for FGIDs and IBS (for more details see Supplementary Methods). Prior to association analysis, microbiome relative abundance data was transformed using center log-ratio (clr) transformation. Taxa (comprising 4 kingdoms, 20 phyla, 36 classes, 62 orders, 150 families, 374 genera and 1117 species; Table S2), pathways (*n* = 606; Table S3) and GBMs (with a minimum coverage of 0.3, *n* = 49; Table S4) were included in the analysis if they were present in more than 5% of participants. In total, 373 species-level taxa, 164 pathways and 36 GBMs were present in more than 5% of samples. Heatmaps were made using the ComplexHeatmap package in R.^42^

#### Association analysis and multiple testing adjustment

Associations of the gut microbiome with each internalizing disorder were assessed in comparison to 5,522 controls, which were defined as participants without any internalizing disorders. Thus, for ‘case – control’ associations of internalizing disorders, a total of 6,013 participants were included in the analysis (Figure 1). All 7,656 participants were included in the single-trait analysis of the gut microbiome with SSRIs or any PTDs, adjusted for baseline covariates (for more details see Supplementary Methods). For all microbiome-related statistical analyses, Benjamini-Hochberg correction was used to adjust for multiple testing. An FDR below 5% was considered statistically significant.

## Supplementary Material

Supplemental Material

## Data Availability

Scripts used for data analysis can be found at: https://github.com/GRONINGEN-MICROBIOME-CENTRE/DMP and https://github.com/GRONINGEN-MICROBIOME-CENTRE/DMP_MentalHeath
