# Supplementary material for: Gut feelings: the relations between depression, anxiety, psychotropic drugs and the gut microbiome
Source: Gut Microbes. 2023 Nov 28;15(2):2281360. doi: 10.1080/19490976.2023.2281360 (PMC10730195; doi:10.1080/19490976.2023.2281360)
Supplement: Supplemental Material [file KGMI_A_2281360_SM1167.zip › Brushett_et_al_suppMethodsResults_Relations_depression_anxiety_psychotropic_drugs_and_gut_microbiome_revision.docx]

**ONLINE ONLY SUPPLEMENTS**

This file includes supplementary methods and results of the main text, including supplementary figures and (a list of the) supplementary tables.

**List of Supplementary Figures**

**Figure S1.** Overview of internalizing disorder comorbidities

**Figure S2.** Principle coordinate analysis depicting the differences in overall bacterial composition in study participants

**Figure S3.** Study population overview

**List of Supplementary Tables**

**Table S1.** Summary statistics of phenotypes

**Table S2.** Summary statistics of gut microbiome taxonomy

**Table S3.** Summary statistics of microbiome pathways

**Table S4.** Summary statistics of Gut–Brain Modules

**Table S5.** Alpha diversity associated with internalizing disorders, SSRI or any psychotropic drugs

**Table S6.** Alpha diversity associated with internalizing disorders adjusted for SSRI

**Table S7.** Alpha diversity associated with internalizing disorders adjusted for any psychotropic drugs

**Table S8.** Beta diversity (species level) variance explained by internalizing disorders (unadjusted or adjusted for selective serotonin-reuptake inhibitors (SSRIs) or any psychotropic drugs (PTDs)), SSRI (unadjusted) or any PTDs (unadjusted)

**Table S9.** Taxa (at all taxonomic levels) associated with internalizing disorders, SSRI or any psychotropic drugs

**Table S10.** Taxa (at all taxonomic levels) associated with internalizing disorders adjusted for SSRI

**Table S11.** Taxa (at all taxonomic levels) associated with internalizing disorders adjusted for any psychotropic drugs

**Table S12.** Validation of significant taxa (at all taxonomic levels) associated with internalizing disorders adjusted for any psychotropic drugs, further adjusted for antibiotic use and diet quality score

**Table S13.** Pathways associated with internalizing disorders, SSRI or any psychotropic drugs

**Table S14.** Pathways associated with internalizing disorders adjusted for SSRI

**Table S15.** Pathways associated with internalizing disorders adjusted for any psychotropic drugs

**Table S16.** Gut–Brain Modules associated with internalizing disorders, SSRI or any psychotropic drugs

**Table S17.** Gut–Brain Modules associated with internalizing disorders adjusted for SSRI

**Table S18.** Gut–Brain Modules associated with internalizing disorders adjusted for any psychotropic drugs

**Table S19.** Validation of significant pathways associated with internalizing disorders adjusted for any psychotropic drugs, further adjusted for antibiotic use and diet quality score

**Table S20.** Validation of significant Gut–Brain Modules associated with internalizing disorders adjusted for any psychotropic drugs, further adjusted for antibiotic use and diet quality score

**Table S21.** Taxa (at all taxonomic levels) associated with internalizing disorders, SSRI or any psychotropic drugs, additionally adjusted for FGIDs and IBS

**Table S22.** Taxa (at all taxonomic levels) associated with internalizing disorders adjusted for SSRI, additionally adjusted for FGIDs and IBS

**Table S23.** Taxa (at all taxonomic levels) associated with internalizing disorders adjusted for any psychotropic drugs, additionally adjusted for FGIDs and IBS

**Table S24.** Validation of significant taxa (at all taxonomic levels) associated with internalizing disorders adjusted for any psychotropic drugs, further adjusted for antibiotic use and diet quality score and additionally adjusted for FGIDs and IBS

**Table S25.** Pathways associated with internalizing disorders, SSRI or any psychotropic drugs, additionally adjusted for FGIDs and IBS

**Table S26.** Pathways associated with internalizing disorders adjusted for SSRI, additionally adjusted for FGIDs and IBS

**Table S27.** Pathways associated with internalizing disorders adjusted for any psychotropic drugs, additionally adjusted for FGIDs and IBS

**Table S28.** Gut–Brain Modules associated with internalizing disorders, SSRI or any psychotropic drugs, additionally adjusted for FGIDs and IBS

**Table S29.** Gut–Brain Modules associated with internalizing disorders adjusted for SSRI, additionally adjusted for FGIDs and IBS

**Table S30.** Gut–Brain Modules associated with internalizing disorders adjusted for any psychotropic drugs, additionally adjusted for FGIDs and IBS

**Table S31.** Validation of significant pathways associated with internalizing disorders adjusted for any psychotropic drugs, further adjusted for antibiotic use and diet quality score and additionally adjusted for FGIDs and IBS

**Table S32.** Validation of significant Gut–Brain Modules associated with internalizing disorders adjusted for any psychotropic drugs, further adjusted for antibiotic use and diet quality score and additionally adjusted for FGIDs and IBS

**SUPPLEMENTARY METHODS**

This section includes additional details on the methodology described in the Methods section of the manuscript.

**Measures and Procedures**

***Stool sample collection, DNA extraction and sequencing, and profiling of microbiome composition and function***

Stool sample collection and the DNA extraction, sequencing and data cleaning steps were described previously[^1^](https://www.zotero.org/google-docs/?N2XH3C). Briefly, following stool sample collection, microbial DNA was isolated with the QIAamp Fast DNA Stool Mini Kit (Qiagen, Germany), according to the manufacturer’s instructions, using the QIAcube (Qiagen) automated sample preparation system. Metagenomic sequencing was performed at Novogene, China using the Illumina HiSeq 2000 platform to generate approximately 8Gb of 150 bp paired-end reads per sample (mean 7.9 gb, st.dev. 1.2 gb). After characterization of metagenomes by deriving taxonomic composition and microbial biochemical pathways, samples with unrealistic microbiome compositions (eukaryotic or viral abundance >25% of the total microbiome content) or low read numbers (total read depth <10 million) were excluded.

**Microbiome analysis**

In the main analyses, to determine the associations between different microbial features (alpha diversity, beta diversity, bacterial taxa, bacterial pathways and Gut–Brain Modules) and internalizing disorders and the use of selective serotonin-reuptake inhibitors (SSRIs) or any psychotropic drugs (PTDs), we applied the following models (all of which were first adjusted for baseline covariates*):

i) internalizing disorder, SSRIs **or** PTDs adjusted for baseline covariates*:

microbial feature ~ baseline covariates + one internalizing disorder **or** SSRIs **or** PTDs

ii) internalizing disorder adjusted for baseline covariates and SSRIs:

microbial feature ~ baseline covariates + one internalizing disorder + SSRIs

iii) internalizing disorder adjusted for baseline covariates and PTDs:

microbial feature ~ baseline covariates + one internalizing disorder + PTDs

iv) SSRIs adjusted for baseline covariates and internalizing disorder

microbial feature ~ baseline covariates + SSRIs + one internalizing disorder

v) PTDs adjusted for baseline covariates and internalizing disorder

microbial feature ~ baseline covariates + PTDs + one internalizing disorder

*Baseline covariates known to influence the gut microbiome[^1,2^](https://www.zotero.org/google-docs/?uWHtww) included technical parameters (metagenomic sequencing batch number, DNA concentration (ng/µl) and read depth), individual characteristics (age, sex and BMI), stool frequency and consistency assessed by Bristol Stool Scale, and the use of PPIs.

After investigating the models above in the main analyses, models were further adjusted for functional gastrointestinal disorders and irritable bowel syndrome to confirm which associations remained after adjustment because people with mental disorders tend to also present with poor gastrointestinal health[^3^](https://www.zotero.org/google-docs/?Dhqk0s).

Benjamini-Hochberg correction was used to adjust for multiple testing across all comparisons, and results were considered significant at a false discovery rate < 0.05.

**SUPPLEMENTARY RESULTS**

This section includes additional results described in the Results section of the manuscript.

**Cohort description**

In this study, 7,656 Lifelines participants were investigated for internalizing disorders. Participants with any depression (AnyDep (n=226)) included both participants with dysthymia (n=70) and major depressive disorder (MDD (n=156), as they were defined to be mutually exclusive in the Lifelines study. Participants with any anxiety (AnyAnx (n=385)) were those reported as having generalized anxiety disorder (GAD (n=339), social phobia (n=70) or panic disorder (n=11)). Participants could be comorbid for different depressive and anxiety disorders, e.g. 71 participants had both MDD and GAD (**Figure S1**). When investigating the intersection size (**Figure S1**), we observed that 491 participants had one or more internalizing disorders. Lastly, in this study, controls were defined as participants without any internalizing disorders, i.e. participants without AnyDep and AnyAnx. Thus, of 7,656 Lifelines participants, 6,013 were available for the case-control section of this study, with 5,522 identified as controls and cases defined as indicated in the schematic of **Figure 1** in the main text. The remaining 1,643 participants were excluded as information was not available for one or more questionnaires.

**
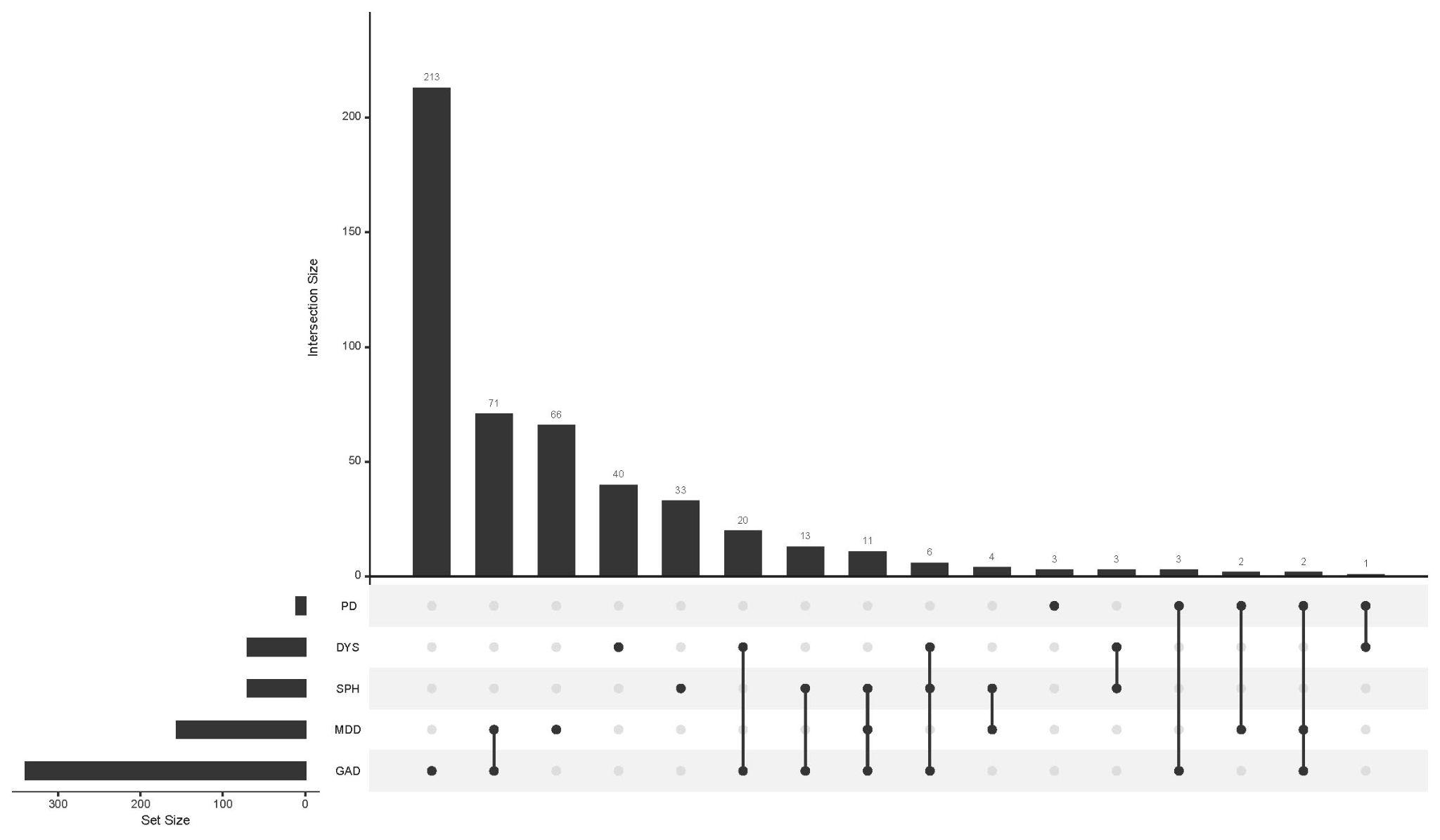
**

**Figure S1. Overview of internalizing disorder comorbidities.** UpSet plot illustrating the overlap between different internalizing disorders in this study. Abbreviations – GAD: generalized anxiety disorder, MDD: major depressive disorder, SPH: social phobia, DYS: dysthymia, PD: panic disorder.

| 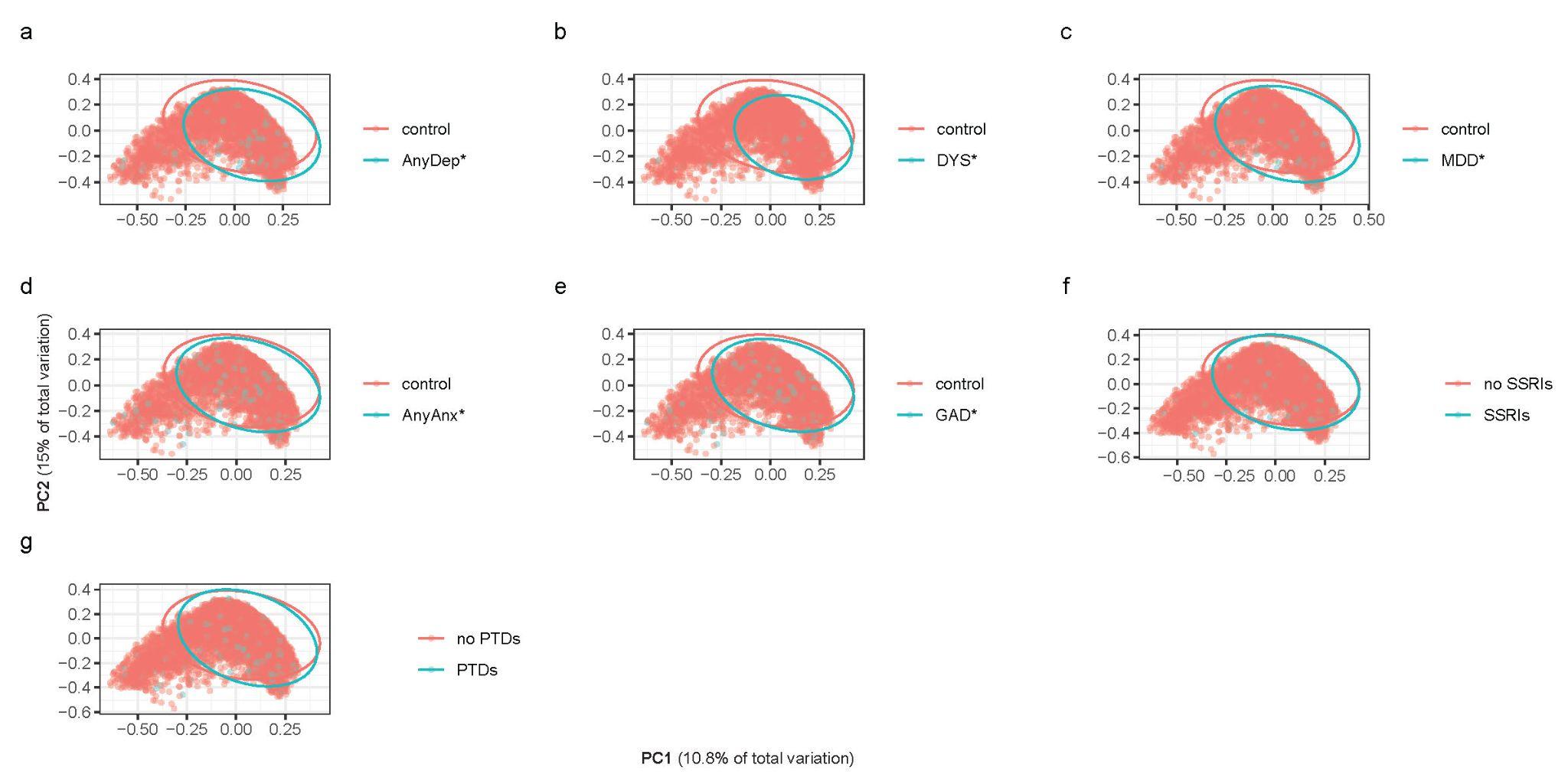 |
| --- |

**Figure S2. Principle coordinate analysis depicting the differences in overall bacterial composition in study participants. a** Any depression (AnyDep; n=226) and control samples (n=5,522). **b** Dysthymia (n=70) and control samples. **c** Major depressive disorder (MDD; n=156) and control samples. **d** Any anxiety (AnyAnx; n=385) and control samples. **e** Generalized anxiety disorder (GAD; n=339) and control samples. **f** Selective serotonin-reuptake inhibitor use (SSRIs=234, no SSRIs=7,422). **g** Any psychotropic drug use, including SSRIs (PTDs =373, no PTDs=7,283). Data points indicate the overall gut microbiome composition of each participant in the study using Bray-Curtis distances. Ellipses indicate the differences between the two groups depicted. * indicates significantly different overall gut microbiome composition (*P*_adj_<0.05).

| **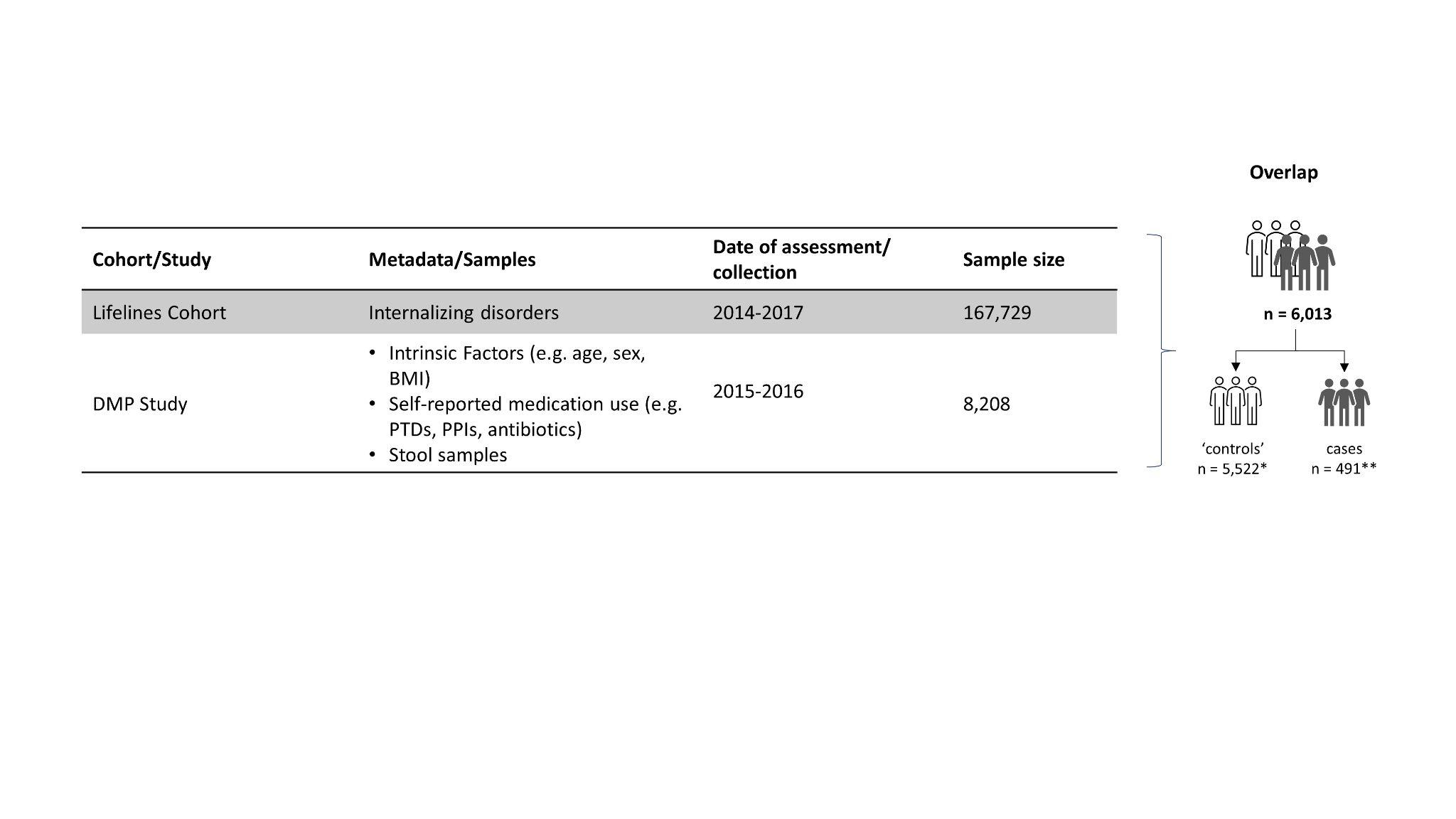Figure S3. Study population overview.** Overview of the overlapping data of participants in this study, which were acquired from two cohorts/studies. Sample sizes of participants with disorders ranged from 70–385 participants, indicated as the *n* in the Figure. Abbreviations – DMP: Dutch microbiome Project[^1^](https://www.zotero.org/google-docs/?HcDeKW), BMI: Body Mass Index, PTD: psychotropic drugs, PPI: proton pump inhibitors. *Controls were defined as participants without any internalizing disorders. **One or more internalizing disorders were observed in 491 participants (see **Figure S1** for more details). |
| --- |

**SUPPLEMENTARY TABLES**

*Supplementary Tables were submitted separately (in Excel format) due to size.*

**References**

[1. Gacesa, R. *et al.* Environmental factors shaping the gut microbiome in a Dutch population *Nature* **604**(7907): 732–739 (2022).](https://www.zotero.org/google-docs/?TqgMPH)

[2. Zhernakova, A. *et al.* Population-based metagenomics analysis reveals markers for gut microbiome composition and diversity. *Science* **352**(6285): 565–9 (2016).](https://www.zotero.org/google-docs/?TqgMPH)

[3. Koloski, N. A. *et al.* The brain–gut pathway in functional gastrointestinal disorders is bidirectional: a 12-year prospective population-based study. *Gut* **61**, 1284–1290 (2012).](https://www.zotero.org/google-docs/?TqgMPH)
